# *MC4R* Single Nucleotide Polymorphisms Were Associated with Metabolically Healthy and Unhealthy Obesity in Chinese Northern Han Populations

**DOI:** 10.1155/2019/4328909

**Published:** 2019-11-06

**Authors:** Luying Gao, Linjie Wang, Hongbo Yang, Hui Pan, Fengying Gong, Huijuan Zhu

**Affiliations:** ^1^Key Laboratory of Endocrinology of National Health Commission, Department of Endocrinology, Peking Union Medical College Hospital, Chinese Academy of Medical Science and Peking Union Medical College, Beijing 100730, China; ^2^Department of Ultrasound, Peking Union Medical College Hospital, Chinese Academy of Medical Science and Peking Union Medical College, Beijing 100730, China

## Abstract

Melanocortin-4 receptor (*MC4R*) has been reported to be associated with the risk of obesity, and metabolically unhealthy obese (MUHO) patients tend to have a greater risk of cardiovascular complications than metabolically healthy obese (MHO) patients. Therefore, we aimed to study single nucleotide polymorphisms (SNPs) in the *MC4R* gene associated with metabolically healthy and unhealthy obesity in Chinese Northern Han populations. A total of 1100 Chinese Northern Han subjects were recruited and divided into four groups according to the criteria of the Adult Treatment Panel-III (ATP-III) and World Health Organization (WHO): MUHO (*n* = 300), MHO (*n* = 196), metabolic unhealthy normal weight (MUH-NW) (*n* = 303), and metabolic healthy normal weight (MH-NW) (*n* = 301). DNA samples were extracted, and six SNPs of the *MC4R* gene, including rs2331841, rs656710, rs17782313, rs571312, rs12970134, and rs11872992, were genotyped with the matrix-assisted laser desorption ionization time-of-flight mass spectrometry (MALDI-TOF MS) method. Among the six SNPs of the *MC4R* gene, rs2331841 (A/G) was the most significant and could account for 0.9% of obesity etiology. Compared with the normal weight group, rs2331841 of the *MC4R* gene was associated with obesity (*P*=0.032). The obesity risk of subjects with the AG genotype in the rs2331841 site was 82% higher than the risk of those with the GG genotype (*β* = 0.60, OR = 1.82, *P*=0.030). After adjusting for sex and age, the frequency of the A allele in the rs2331841 site was higher in the MUHO group than in the MH-NW group (27.9% vs. 21.1%, respectively, OR = 1.49, 95% CI 1.14–1.96, *P*=0.005) and in the MUHO group than in the MHO group (27.9% vs. 22.3%, respectively, OR = 1.39, 95% CI 1.02–1.92, *P*=0.039). Among the three genotypes of rs2331841, the subjects with the AA/AG genotype had higher diastolic blood pressure (DBP) than those with the GG genotype. Our data first suggest that SNPs in the rs2331841 site of the *MC4R* gene are closely related to obesity and its related metabolic disorders in Chinese Northern Han populations. The participants with an A allele of rs2331841 had a higher risk of obesity and MUHO than other participants.

## 1. Introduction

The global incidence of obesity is increasing yearly with increased socioeconomic development. Obesity is the main risk factor for type 2 diabetes mellitus, hypertension, hyperlipidemia, and coronary heart disease, and it is a common disease and threatens human health worldwide [1]. However, not every obese patient is subjected to these comorbidities. According to the criteria of the Adult Treatment Panel-III (ATP-III), obesity includes metabolically unhealthy obesity (MUHO) and metabolically healthy obesity (MHO). MHO patients tend to have less risk for cardiovascular and other complications in comparison with MUHO patients, and these two types of obesity have different features in their etiologies and treatments [2, 3].

Obesity is a multifactorial disease. A twin study showed that intrapair correlation coefficients of body mass index (BMI) between monozygotic twin pairs were higher than those between dizygotic twin pairs [4], which indicated that genetic factors played an important role in pathogenicity. Recently, melanocortin-4 receptor (*MC4R*) has been reported to be associated with the risk of obesity by the Genome-Wide Association Study (GWAS) [5]. The single nucleotide polymorphisms (SNPs) at rs12970134, rs17782313, rs571312, rs2331841, rs6567160, and rs11872992 in/near the melanocortin-4 receptor (*MC4R*) gene have been associated with lower energy expenditure in overweight and obese children and adults [6–9]. However, the association between the genetic variations of the *MC4R* gene and obesity with different metabolic abnormalities is still not fully elucidated. Therefore, in the present study, we aimed to investigate the association of SNPs of the *MC4R* gene with metabolically healthy and unhealthy obesity in Chinese Northern Han populations.

## 2. Methods

### 2.1. Study Design and the Characteristics of the Subjects

In total, 1100 Northern Han Chinese (18–83 y) who were long-term residents of Beijing were recruited. All the subjects were independently recruited from the Endocrine Clinic and Physical Examination Center at the Peking Union Medical College Hospital in Beijing. The following clinical information and biochemical parameters were measured and collected: body weight, height, BMI, FBG (fasting blood glucose), TC (total cholesterol), SBP (systolic blood pressure), DBP (diastolic blood pressure), HDL (high-density lipoprotein) cholesterol, LDL (low-density lipoprotein) cholesterol, TG (triglycerides), BUN (blood urea nitrogen), UA (uric acid), ALT (alanine aminotransferase), AST (aspartate transaminase), and Cr (creatine). Obesity was defined as BMI ≥30 kg/m^2^, and BMI ≤25 kg/m^2^ was considered normal weight based on the criteria of the World Health Organization (WHO). The obesity group and normal weight group were then subdivided into metabolically healthy and metabolically unhealthy subgroups based on the criteria of the Adult Treatment Panel-III (ATP-III) (high triglycerides (≥1.7 mmol/L), elevated SBP (≥130 mmHg) or DBP (≥85 mmHg), higher FBP (≥5.6 mmol/L), and lower HDL-cholesterol (1.04 mmol/L for men and 1.29 mmol/L for women)). Metabolically healthy subjects were defined as having less than two of the metabolic abnormalities in the ATP-III criteria, while metabolically abnormal patients had at least two abnormalities. Therefore, a total of 1100 participants were divided into the following four groups: metabolically unhealthy obesity (MUHO, BMI ≥30 kg/m^2^ and ATP-III ≥2), metabolic healthy obesity (MHO, BMI ≥30 kg/m^2^ and ATP-III ≤1), metabolic unhealthy normal weight (MUH-NW, BMI ≤ 25 kg/m^2^ and ATP-III ≥2), and metabolic healthy normal weight (MH-NW, BMI ≤25 kg/m^2^ and ATP-III ≤1). Age, sex, and BMI were prematched within the two normal weight subgroups and the two obesity subgroups as presented in Table 1.

Any patient with either hepatic or renal dysfunction was excluded in the present study. Blood samples were collected for DNA extraction and further genotyping. Informed consent was obtained from each patient. This study was conducted in accordance with the Declaration of Helsinki, and the study protocol was approved by the Ethics Committee of Peking Union Medical College Hospital (No. S-364).

### 2.2. Selection of SNPs and Genotyping

According to the minor allele frequency (MAF), more than 5% in the Chinese population (http://browser.1000genomes.org/index.html), the heterozygosity and the location in the *MC4R* gene, six SNPs, including rs2331841 (A/G), rs656710 (C/T), rs17782313 (C/T), rs571312 (A/C), rs12970134 (A/G), and rs11872992 (A/G), were selected for further association study in our total of 1100 DNA samples.

Genomic DNA was extracted from peripheral white blood cells using a DNA extraction kit (Omega Biotek, GA, USA) according to the manufacturer's instructions. A Mass ARRAY MALDI-TOF system (Sequenom Inc., San Diego, CA, USA) was used for genotyping the candidate SNPs. Hardy–Weinberg equilibrium was assessed by the exact test using Plink software (version 1.06, http://pngu.mgh.harvard.edu/purcell/plink).

### 2.3. Statistical Analyses

Quantitative data are presented as the mean ± standard deviation (SD). The chi-squared test with Yates' correction and Fisher's exact test were used to compare the differences in allele frequencies and distributions of genotypes between the cases and controls. The relationship between the studied variables was evaluated by one-way analysis of variance (ANOVA). The above analyses were performed with SPSS 17.0 software (SPSS, Inc., Chicago, Illinois). The Hardy–Weinberg equilibrium test was performed using Pearson's chi-squared test with Plink. Genotype and allele frequencies for the case and control groups were compared by calculating the odds ratios (ORs) and their 95% confidence intervals (95% CIs) with logistic regression, which was performed with Plink (version 1.06, http://pngu.mgh.harvard.edu/purcell/plink). Linkage disequilibrium statistics were computed using *D*′ and *R*^2^, tested with Haploview (version 4.1, http://www.broadinstitute.org), and haplotype frequencies were estimated using Haploview. *P* values were adjusted by the false discovery rate method, and *P* < 0.05 was considered statistically significant.

## 3. Results

### 3.1. Clinical Characteristics of the Study Subjects

Demographic and clinical data for all participants are shown in Table 1. Baselines for the obese patients vs. the normal weight patients and MUHO vs. MH-NW were matched with respect to age and sex. There was no significant difference in BMI between the two normal weight subgroups and the two obesity subgroups. As we expected, participants in the obese group had higher BMIs, weight, SBP, DBP, FBG, TG, and LDL levels and lower HDL levels than those in the normal weight group (all *P* < 0.01). Certainly, subjects in the MUHO group also had higher BMIs, weight, SBP, DBP, FBP, TG, and TC and lower HDL levels than those in the MH-NW group.

As presented in [Supplementary-material supplementary-material-1], the average genotype call rate was 99.8%. Minor allele frequencies (MAF) of the six SNPs of the *MC4R* gene in the obese and normal weight groups were similar to the MAF of Han Chinese in Beijing (CHB) (http://browser.1000genomes.org/). All SNPs were in Hardy–Weinberg equilibrium and had *P* values greater than 0.05, as presented in [Supplementary-material supplementary-material-1].

### 3.2. Comparison between the Obesity and Normal Weight Groups

The genotypes and allele frequencies of the six SNPs in the *MC4R* gene in the obesity and normal weight groups were compared. As shown in Table 2, after adjusting for sex and age, a significant difference was found in the genotype frequencies of the rs233184 (A/G) polymorphism between the obesity and normal weight groups (0.07/0.36/0.57 vs. 0.04/0.35/0.61, respectively, *P*=0.032), but this difference was not statistically significant after the false discovery rate (FDR) correction (*P* < 0.05). Further multivariate logistic regression analysis with recessive inheritance models (GG + AG vs. AA) were performed, and the results showed that rs2331841 was still significantly associated with obesity (OR = 1.84, *P*=0.039), as presented in Table 3.

Next, the effect of a single SNP on obesity was estimated by logistic regression. As shown in Table 4, the obesity risk of subjects with the AG genotype at the rs233184 site was 82% higher than that of those with the GG genotype (*β* = 0.60, OR = 1.82, *P*=0.030). The obesity risk of subjects with the AA genotype was 28% higher than that of those with the GG genotype, although the *P* value was 0.058 (*β* = 0.25, OR = 1.28, *P*=0.058). Finally, the results further showed that rs233184 accounted for 0.9% of obesity etiology (Nagelkerke *R*^2^ = 0.009).

### 3.3. Comparison between the Obesity and Metabolic Healthy Normal Weight (MH-NW) Groups

In the obesity and metabolic healthy normal weight (MH-NW) groups, rs233184 still showed statistically significant associations with obesity. The frequency of the A allele of rs2331841 was higher in the obese group than in the MH-NW group (25.7% vs. 21.1%), and the A allele of rs2331841 was associated with an increased risk of obesity with a per-allele OR of 1.32 (95% CI 1.02–1.69; *P*=0.034) after adjusting for sex and age, as presented in Table 2.

### 3.4. Comparison between the Metabolically Unhealthy Obesity (MUHO) and Metabolic Healthy Normal Weight (MHO) Groups

Allele and genotype frequency association analyses were also performed for the metabolically unhealthy obesity (MUHO) and the metabolic healthy normal weight (MH-NW) groups. As shown in Table 2, four of the 6 SNPs, including rs2331841, rs656710, rs17782313, and rs571312, showed statistically significant associations with MUHO when compared with MH-NW. After adjusting for sex and age, the frequency of the A allele in the rs2331841 site was higher in the MUHO group than in the MH-NW group (27.9% vs. 21.1%, respectively, OR = 1.49, 95% CI 1.14–1.96, *P*=0.005); the frequency of the C allele in the rs6567160 site was higher in the MUHO group than in the MH-NW group (26.6% vs. 20.6%, respectively, OR = 1.45, 95% CI 1.09–1.92, *P*=0.001); the frequency of the A allele in the rs571312 site was higher in the MUHO group than in the MH-NW group (26.2% vs. 20.7%, respectively, OR = 1.43, 95% CI 1.08–1.89, *P*=0.013); and the frequency of the C allele in the rs17782313 site was higher in the MUHO group than in the MH-NW group (26.3% vs. 20.4%, respectively, OR = 1.41, 95% CI 1.06–1.67, *P*=0.015). Importantly, all these findings were still statistically significant after the FDR correction, as shown in Table 2 (all *P* < 0.05).

As shown in Table 3, the four SNPs, rs2331841, rs656710, rs17782313, and rs571312, were significantly associated with MUHO with the dominant model (OR = 1.43∼1.49; *P*=0.019 ~ 0.039), and two SNPs, rs2331841 and rs656710, were significantly associated with MUHO with the recessive model (OR = 2.38, *P*=0.017; OR = 2.12, *P*=0.049) after adjusting for sex and age.

### 3.5. Comparison between the Metabolically Unhealthy Obesity (MUHO) and Metabolically Healthy Obesity (MHO) Groups

The same allele and genotype frequency association analyses were performed between the metabolically unhealthy obesity (MUHO) and metabolically healthy obesity (MHO) groups. The results demonstrated that SNPs in the rs233184 site were statistically associated with MUHO after adjusting for sex and age, and the frequency of the A allele of rs2331841 was higher in the MUHO group than in the MHO group (27.9% vs. 22.3%, respectively, OR = 1.39, 95% CI 1.02–1.92, *P*=0.039). However, it did not remain statistically significant after the FDR correction. Furthermore, there were significant differences in three genotype frequencies in rs2331841, rs17782313, and rs12970134 sites, as presented in Table 2 (all *P* < 0.05). As shown in Table 3, the genotypes of the rs2331841 and rs12970134 sites were both significantly associated with MUHO with the recessive inheritance models (OR = 2.93, *P*=0.022, and OR = 3.98, *P*=0.030, respectively).

### 3.6. Haplotype Association of *MC4R* with Obesity

**Figure 1:**
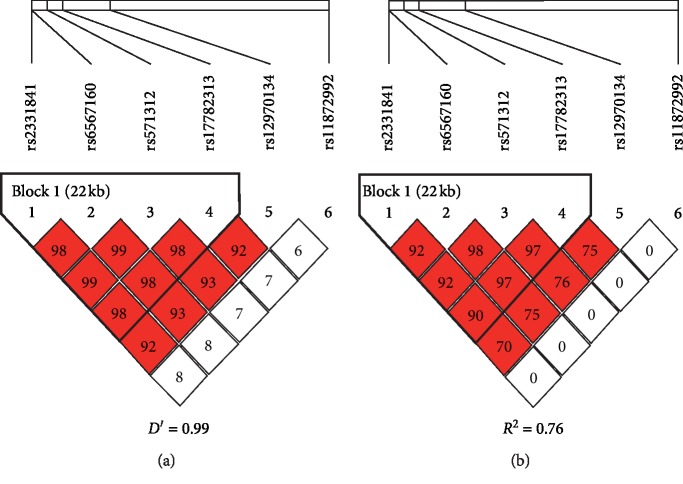
Linkage disequilibrium blocks of *MC4R* gene. (a) *D*′ = 0.99. (b) *R*^2^ = 0.76.

As shown in Figure 1, the linkage disequilibrium (LD) analysis for the six SNPs in the *MC4R* gene demonstrated that rs2331841, rs6567160, rs571312, and rs17782313 were in one linkage disequilibrium region. rs2331841, rs6567160, rs571312, and rs17782313 belonged to the same block, indicating that they were in very strong linkage disequilibrium (*D*′ = 0.99, *R*^2^ = 0.76).

Further haplotype analysis of these four SNPs of the *MC4R* gene (rs2331841, rs6567160, rs571312, and rs17782313) in the normal weight and obese subjects revealed three constructed haplotypes. As presented in Table 5, the constructed haplotype ACAC of these four SNPs was significantly associated with obesity (23% vs. 21%, *P*=0.01). However, the most common haplotype GTCT was not significantly associated with obesity (76% vs. 78%, *P*=0.12).

### 3.7. Association of Different Genotypes in *MC4R* with Obesity-Related Traits

To further investigate the association of different genotypes of *MC4R* with obesity-related traits, a total of 1100 individuals were recruited, and a one-way ANOVA was used. As presented in Table 6, there was a significant difference in diastolic blood pressure (DBP) among the three genotypes of rs2331841 (*P*=0.02), rs6567160 (*P*=0.01), rs571312 (*P*=0.01), rs17782313 (*P*=0.02), and rs12970134 (*P*=0.03). Further analysis found that the DBP of individuals with AA genotypes in the rs2331841 site was significantly higher than that of those with AG/GG genotypes (*P*=0.03). Accordingly, individuals with TT genotypes for the rs17782313 site, CC genotypes for the rs571312 site, GG genotypes for the rs12970134 site, and TT genotypes for the rs6567160 site all had lower DBP than those with CT/CC genotypes for the rs17782313 site, AC/AA genotypes for the rs571312 site, AG/AA genotypes for the rs12970134 site, and CT/CC genotypes for the rs6567160 site, respectively (all *P* < 0.05). Moreover, associations between MC4R variants and DBP were evaluated by the logistic regression after adjusting BMI, age, and sex. The results showed that rs17782313 SNPs, rs571312 SNPs, rs11872992 SNPs, and rs6567160 SNPs were still statistically associated with DBP (OR = 0.055, 95% CI 0.016–2.26, *P*=0.047 for rs17782313 SNPs; OR = −0.059, 95% CI −2.37 to 0.1, *P*=0.033 for rs571312 SNPs; OR = −0.060, 95% CI −2.37 to 0.12, *P*=0.030 for rs6567160 SNPs), while rs2331841 SNPs, rs12970134 SNPs, and rs11872992 SNPs were not statistically associated with DBP (OR = −0.052, 95% CI −2.17 to 0.041, *P*=0.059 for rs2331841 SNPs; OR = −0.040, 95% CI −2.07 to 0.32, *P*=0.15 for rs12970134 SNPs; rs11872992 (OR = 0.006, 95% CI −1.00 to 1.23, *P*=0.83). However, other anthropometric and metabolic parameters, including BMI, weight, SBP, FBG, TG, HDL, TC, and LDL, were not significantly different among the three genotypes of the six SNPs, as presented in [Supplementary-material supplementary-material-1].

## 4. Discussion

The *MC4R* (melanocortin-4 receptor) gene is located on chromosome 18. Mutations in the *MC4R* gene were found at a frequency of approximately 3-4% in severe early-onset obesity, and the *MC4R* mutation is the most common monogenic cause of obesity, with a prevalence of 1.7–3.0% in obese individuals [10–12]. Obesity is further subdivided into metabolically unhealthy obesity and metabolically healthy obesity according to the patient's metabolic features. Compared with MHO, metabolically unhealthy obese patients tend to have more risk for cardiovascular complications and all-cause death. The fact that some obese individuals enjoy metabolic health or an unhealthy status could be due to genetic predisposition [2, 3]. Moreover, recent studies found that the SNPs rs12970134, rs17782313, rs571312, rs2331841, rs6567160, and rs11872992 in the *MC4R* gene were associated with obesity or obesity-related parameters [6–9].

Therefore, in the present study, we first found that rs2331841 (A/G) accounted for 0.9% of obesity etiology. The genotype frequency in rs2331841 of obese patients and normal weight subjects showed that A-allele carriers have an increased susceptibility to obesity when compared with the G-allele carriers. Further logistic regression analysis with recessive inheritance models (GG + AG vs. AA) showed that rs2331841 was still significantly associated with obesity and the obesity risk of the AG genotype was much higher than that of the GG genotype. Moreover, the constructed haplotype ACAC of four SNPs of the *MC4R* gene (rs2331841, rs6567160, rs571312, and rs17782313) was significantly associated with obesity. These findings were consistent with a previous GWAS performed in 62,245 East Asian subjects, which demonstrated that rs2331841 was significantly associated with BMI [13]. In addition, a recent study performed in 3,506 Chinese children and adolescents also showed that rs2331841 was robustly associated with childhood obesity [14]. Interestingly, our study further found that rs2331841 increased the genetic risks of metabolic unhealthy obesity because a significant positive association in both allelic and genotypic frequency was still found between the MUHO group and the MHO group in addition to the MUHO group and the MH-NW group. These results suggest that rs2331841 SNPs are not only associated with obesity but also associated with metabolic disorders in obese individuals. Moreover, we found that individuals with AA genotypes in the rs2331841 site had significantly higher DBP than those with AG/GG genotypes. However, the associations between rs2331841 SNPs and blood pressure were not significant after adjusting BMI, age, and sex as covariates (*P*=0.059), suggesting that the associations between rs2331841 SNPs and DBP may be partly due to obesity. These results suggest that rs2331841 is significantly associated with obesity and its related comorbidity and that it may play an important role in the development of obesity and its related metabolic disorders.

Allele and genotype frequency association analysis between the MUHO and the MH-NW groups showed that C-allele carriers in the rs17782313 site have an increased susceptibility to MUHO compared to the T-allele carriers and that rs17782313 was significantly associated with MUHO under the dominant model. In agreement with our results, Huang et al. found that rs17782313 was strongly associated with obesity and BMI in Chinese Southern Han populations [15]. A systematic review and meta-analysis including 49 studies reported that the rs17782313 C allele was strongly associated with obesity (OR = 1.18, 95% CI = 1.15–1.2) [16]. In addition, our study demonstrated that diastolic blood pressure was different among the three genotypes in rs17782313 in all subjects and was independent of obesity. The association of rs17782313 with blood pressure was also observed in patients with hypertension [17].

Next, three SNPs, rs571312 (C > A), rs12970134 (G > A), and rs6567160 (T > C), were found to be associated with MUHO when compared with the MH-NW group. Moreover, a meta-analysis including seven studies reported that rs12970134 and rs571312 were significantly associated with obesity in 27,715 East Asians (OR = 1.12, 95% CI = 1.08–1.15 and OR = 1.19, 95% CI = 1.10–1.29, respectively) [18]. A meta-analysis performed in East Asians reported that the rs571312 A allele increased the risk of obesity (OR = 1.19, 95% CI = 1.10–1.29) [19] and that rs571312 was associated with BMI among Singaporean Chinese, Malay, and Asian-Indian populations and pediatric obesity populations [20, 21]. Together, these results suggest that rs571312, rs12970134, and rs6567160 are significantly associated with obesity and metabolism, especially with metabolic disorders in obese individuals.

There are increasing numbers of studies on the types of obesity, namely, MHO and MUHO. MHO seems to have a protective mechanism. Compared with MUHO, MHO has different features regarding body fat distribution, the inflammation spectrum, metabolism, and weight loss [3], and *MC4R* gene polymorphism may be involved. The mechanisms of how *MC4R* gene polymorphism influences MUHO are unclear. The melanocortin pathway in the human hypothalamus plays a pivotal role in regulating food intake and mediating leptin and energy homeostasis. Studies show that MUHO patients have increased leptin levels [22]. Adipose tissue secretes leptin in response to increased fat storage, which circulates as an afferent satiety signal and activates hypothalamic neurons expressing POMC located in the arcuate nucleus [23]. POMC is a polypeptide that undergoes tissue-specific posttranslational processing, the products of which include the melanocortin peptides *α*, *β*, and *γ*-melanocyte-stimulating hormones (MSH) [24], and 1 or more of the 3 melanocortin peptides are involved in the anorectic response (i.e., reduced feeding) by stimulating melanocortin-4 receptors (*MC4R*) on neurons downstream in the paraventricular nucleus (PVN) [25]. Leptin may be responsible for the effect of the *MC4R* gene on MUHO. Moreover, the POMC-MC4R system regulates blood pressure in obesity [26]. Melanocortinergic signaling controls human blood pressure through an insulin-independent mechanism [27, 28].

## 5. Conclusions

Our present study first demonstrated that *MC4R* SNPs (rs2331841, rs6567160, rs17782313, rs571312, and rs12970134) are associated with obesity, especially for metabolic disorders in obese individuals. A risk allele and an AA genotype of rs2331841 (MC4R) could increase the risk of obesity. The participants with the A risk allele of rs2331841 had a higher risk of MUHO than other participants. The participants with low-frequency alleles of rs656710, rs17782313, rs571312, and rs12970134 (*MC4R*) had a higher risk of MUHO than other participants. Genetic analysis may be useful to identify high-risk participants who may need preventive interventions to reduce the incidence of obesity-related metabolic disorders.

## Figures and Tables

**Table 1:** Demographic and clinical data of all participants.

Traits	Obesity			Normal weight			*P* ^*∗*^	*P* ^#^	*P* ^Δ^	*P* ^*▲*^
	Obesity	MUHO	MHO	Normal weight	MUH-NW	MH-NW				
*N*	496	300	196	604	303	301				
Male/female	262/234	150/150	112/84	306/298	144/159	162/139	0.86	0.47	0.11	0.08
Age (years)	46.6 ± 11.9	47.7 ± 12.5	44.8 ± 12.6	46.8 ± 11.4	48.3 ± 11.4	45.2 ± 10.9	0.16	0.08	0.09	0.16
BMI (kg/m2)	32.5 ± 3.2	32.7 ± 3.9	32.2 ± 2.8	22.6 ± 1.6	23.1 ± 1.8	22.1 ± 1.7	<0.01	<0.01	0.06	0.19
Height (cm)	163.8 ± 8.8	164.6 ± 8.8	162.7 ± 8.5	164.9 ± 9.0	164.8 ± 8.6	165.1 ± 9.8	0.06	0.51	0.05	0.39
WC (cm)	99.9 ± 9.6	101.2 ± 10.0	98.0 ± 8.0	80.5 ± 7.4	83.3 ± 7.0	77.6 ± 7.2	<0.01	<0.01	0.90	0.33
Weight (kg)	87.8 ± 17.7	89.4 ± 17.2	85.3 ± 12.0	62.1 ± 8.6	62.9 ± 8.6	61.2 ± 10.1	<0.01	<0.01	<0.01	<0.01
DBP (mmHg)	131.9 ± 18.2	136.0 ± 19.0	125.6 ± 15.3	118.4 ± 15.6	123.5 ± 17.7	113.3 ± 10.9	<0.01	<0.01	0.01	<0.01
SBP (mmHg)	84.0 ± 12.2	86.0 ± 12.7	80.9 ± 11.5	75.7 ± 10.3	79.2 ± 11.5	72.1 ± 7.8	<0.01	<0.01	<0.01	<0.01
FBG (mmol/L)	6.9 ± 1.3	6.2 ± 1.6	5.4 ± 1.0	5.5 ± 1.6	5.9 ± 1.6	5.0 ± 0.4	<0.01	<0.01	<0.01	<0.01
TG (mmol/L)	2.0 ± 1.5	2.4 ± 1.7	1.4 ± 0.6	1.7 ± 1.5	2.3 ± 1.9	1.1 ± 0.8	<0.01	<0.01	<0.01	0.08
HDL (mmol/L)	1.3 ± 0.5	1.1 ± 0.3	1.6 ± 0.8	1.3 ± 0.3	1.2 ± 0.3	1.5 ± 0.4	<0.01	<0.01	0.09	<0.01
TC (mmol/L)	4.9 ± 1.0	4.9 ± 1.0	4.9 ± 0.9	4.9 ± 1.0	5.0 ± 1.0	4.8 ± 0.9	0.05	0.046	0.46	<0.01
LDL (mmol/L)	3.1 ± 0.8	3.1 ± 0.8	3.1 ± 0.8	3.0 ± 0.8	3.1 ± 0.8	2.9 ± 0.7	<0.01	0.032	0.06	0.19

*P* ^*∗*^, OB vs. NW; *P*^#^, MUHO vs. MH-NW; *P*^Δ^, MUHO vs. MHO; *P*^*▲*^, MUH-NW vs MH-NW. MUH-NW, metabolic unhealthy normal weight; MH-NW, metabolic healthy normal weight; MUHO, metabolically unhealthy obesity; MHO, metabolically healthy obesity; BMI, body mass index; WC, waist circumference; SBP, systolic blood pressure; DBP, diastolic blood pressure; FBG, fasting blood glucose; HDL-C, high-density lipoprotein; TG, triglyceride; LDL, low-density lipoprotein; TC, total cholesterol. Data were expressed by mean ± standard deviation.

**Table 2:** Allelic and genotypic association of SNPs in the case-control study using logistic regression.

SNPs	Allele	Allele gene				Genotype count			
	1/2	1/2	1/2	OR	*P*	11/12/22	11/12/22	OR	*P*
		Case	Control	(95% CI)		Case	Control	(95% CI)	
Obesity: normal weight									
rs2331841	A/G	276/842	198/722	1.21 (0.98, 1.50)	**0.07**	38/200/321	18/162/280	1.38 (1.03, 1.85)	**0.032**
rs6567160	C/T	262/858	193/729	1.16 (0.94, 1.44)	0.17	33/196/331	17/159/285	1.30 (0.96, 1.77)	0.089
rs571312	A/C	259/859	189/725	1.16 (0.94, 1.44)	0.17	32/195/332	17/155/285	1.28 (0.94, 1.73)	0.12
rs17782313	C/T	259/859	193/727	1.14 (0.92, 1.41)	0.22	34/191/334	18/157/285	1.28 (0.95, 1.72)	0.11
rs12970134	A/G	228/888	180/742	1.07 (0.86, 1.34)	0.55	24/180/354	11/158/292	1.37 (0.95, 1.98)	0.093
rs11872992	A/G	233/887	207/713	0.90 (0.73, 1.11)	0.31	32/169/359	24/159/277	1.00 (0.76, 1.32)	0.99

Obesity: metabolic healthy normal weight (MH-NW)									
rs2331841	A/G	234/676	120/450	1.32 (1.02, 1.69)	**0.034**	31/172/252	12/96/177	1.35 (1.92, 0.96)	**0.08**
rs6567160	C/T	224/686	118/454	1.27 (0.98, 1.64)	0.065	27/170/258	11/96/179	1.32 (0.92, 1.89)	0.14
rs571312	A/C	220/686	116/452	1.27 (0.98, 1.64)	0.074	26/168/259	11/94/179	1.28 (0.89, 1.92)	0.17
rs17782313	C/T	223/687	118/452	1.25 (0.97, 1.61)	0.079	28/167/260	12/94/179	1.28 (0.90, 1.82)	0.17
rs12970134	A/G	196/714	115/457	1.10 (0.85, 1.43)	0.47	20/156/279	8/99/179	1.28 (0.84, 1.96)	0.25
rs11872992	A/G	201/709	123/447	1.02 (0.79, 1.30)	0.89	30/141/284	11/101/173	1.27 (0.88, 1.82)	0.2

Metabolically unhealthy obesity (MUHO): metabolic healthy normal weight (MH-NW)									
rs2331841	A/G	156/404	120/450	1.49 (1.14, 1.96)	**0.005 ** ^a^	25/106/149	12/96/177	1.64 (1.14, 2.38)	**0.008**
rs6567160	C/T	149/411	118/454	1.45 (1.09, 1.92)	**0.011 ** ^a^	21/107/152	11/96/179	1.54 (1.05, 2.27)	**0.026**
rs571312	A/C	147/413	116/452	1.43 (1.08, 1.89)	**0.013 ** ^a^	20/107/153	11/94/179	1.49 (1.02, 2.22)	**0.041**
rs17782313	C/T	148/412	118/452	1.41 (1.06, 1.67)	**0.015 ** ^a^	22/104/154	12/94/179	1.52 (1.04, 2.17)	**0.03**
rs12970134	A/G	131/429	115/457	1.25 (0.93, 1.67)	0.14	17/97/166	8/99/179	1.56 (1.01, 2.48)	**0.048**
rs11872992	A/G	117/443	123/447	0.93 (0.70, 1.23)	0.61	17/83/180	11/101/173	1.18 (0.79, 1.75)	0.43

Metabolically unhealthy obesity (MUHO): metabolically healthy obesity (MHO)									
rs2331841	A/G	78/272	156/404	1.39 (1.02, 1.92)	**0.039**	6/66/103	25/106/149	1.75 (1.10, 2.78)	**0.017**
rs6567160	C/T	75/275	149/411	1.37 (0.99, 1.89)	0.056	6/63/106	21/107/152	1.56 (0.98, 2.50)	0.052
rs571312	A/C	73/273	147/413	1.36 (0.98, 1.89)	0.063	6/61/106	20/107/153	1.52 (0.95, 2.44)	0.078
rs17782313	C/T	75/275	148/412	1.36 (0.98, 1.87)	0.065	6/63/106	22/104/154	1.59 (0.99, 2.56)	**0.044**
rs12970134	A/G	65/285	131/429	1.37 (0.97, 1.92)	0.070	3/59/113	17/97/166	1.96 (1.05, 3.67)	**0.027**
rs11872992	A/G	84/266	117/443	0.84 (0.62, 1.14)	0.27	13/58/104	17/83/180	0.87 (0.59, 1.078)	0.36

^a^Remain significant after false discovery rate (FDR) analysis.

**Table 3:** Dominant and recessive model association of SNPs in the case-control study by logistic regression analysis.

SNPs	Allele	Dominant model				Recessive model			
	1/2	11 + 12/22	11 + 12/22	OR	*P*	11/12 + 22	11/12 + 22	OR	*P*
		Case	Control	(95% CI)		Case	Control	(95% CI)	
Obesity: normal weight									
rs2331841	A/G	238/321	180/280	1.18 (0.91, 1.52)	0.21	38/521	18/442	1.84 (1.03, 3.28)	**0.039**
rs6567160	C/T	229/331	176/285	1.13 (0.87, 1.46)	0.35	33/527	17/444	1.66 (0.91, 3.03)	0.099
rs571312	A/C	227/332	172/285	1.14 (0.88, 1.47)	0.32	32/527	17/440	1.58 (0.86, 2.90)	0.14
rs17782313	C/T	225/334	175/285	1.10 (0.86, 1.43)	0.44	34/525	18/442	1.61 (0.89, 2.90)	0.11
rs12970134	A/G	204/354	169/292	1.00 (0.77, 1.30)	0.98	24/534	11/450	1.92 (0.92, 3.98)	0.081
rs1187299	A/G	201/359	183/277	0.84 (0.65, 1.08)	0.17	32/528	24/436	1.07 (0.62, 1.86)	0.8

Obesity: metabolic healthy normal weight (MH-NW)									
rs2331841	A/G	203/252	108/177	1.33 (0.99, 1.82)	0.059	31/424	12/273	1.69 (0.85, 3.33)	0.13
rs6567160	C/T	197/258	107/179	1.30 (0.95, 1.75)	0.099	27/428	11/275	1.59 (0.78, 3.23)	0.2
rs571312	A/C	194/259	105/179	1.30 (0.95, 1.75)	0.1	26/427	11/273	1.52 (0.74, 3.13)	0.25
rs17782313	C/T	195/260	106/179	1.28 (0.94, 1.75)	0.11	28/427	12/273	1.52 (0.75, 3.03)	0.25
rs12970134	A/G	176/279	107/179	1.06 (0.78, 1.45)	0.7	20/435	8/278	1.62 (0.70, 3.70)	0.25
rs11872992	A/G	171/284	112/173	0.92 (0.68, 1.25)	0.59	30/425	11/274	1.69 (0.83, 3.45)	0.14

Metabolically unhealthy obesity (MUHO): metabolic healthy normal weight (MH-NW)									
rs2331841	A/G	131/149	108/177	1.49 (1.06, 2.08)	**0.019**	25/255	12/273	2.38 (1.16, 4.76)	**0.017**
rs6567160	C/T	128/152	107/179	1.45 (1.04, 2.04)	**0.028**	21/259	11/275	2.12 (1.01, 4.54)	**0.049**
rs571312	A/C	127/153	105/179	1.47 (1.04, 2.04)	**0.029**	20/260	11/273	2.04 (0.93, 4.17)	0.078
rs17782313	C/T	126/154	106/179	1.43 (1.02, 2.00)	**0.039**	22/258	12/273	2.04 (0.98, 4.17)	0.057
rs12970134	A/G	114/166	107/179	1.18 (0.83, 1.64)	0.36	17/263	8/278	2.32 (0.99, 5.56)	0.053
rs11872992	A/G	100/180	112/173	0.83 (0.58, 1.16)	0.28	17/263	11/274	1.52 (0.69, 3.33)	0.3

Metabolically unhealthy obesity (MUHO): metabolically healthy obesity (MHO)									
rs2331841	A/G	131/149	72/103	1.31 (0.89, 1.93)	**0.07**	25/255	6/169	2.93 (1.17, 7.35)	**0.022**
rs6567160	C/T	128/152	69/106	1.35 (0.91, 1.97)	0.14	21/259	6/169	2.37 (0.93, 6.05)	0.07
rs571312	A/C	127/153	67/106	1.35 (0.91, 1.99)	0.13	20/260	6/167	2.15 (0.84, 5.51)	0.11
rs17782313	C/T	126/154	69/106	1.30 (0.88, 1.92)	0.18	22/258	6/169	2.48 (0.98, 6.28)	0.056
rs12970134	A/G	114/166	62/113	1.27 (0.85, 1.88)	0.24	17/263	3/172	3.98 (1.14, 13.89)	0.030
rs11872992	A/G	100/180	71/104	0.82 (0.55, 1.21)	0.32	17/263	13/162	0.74 (0.35, 1.57)	0.43

**Table 4:** Logistic regression of SNPs with obese and normal weight subjects.

rs2331841	*β*	SE	Walds	*P*	OR
GG			7.07	0.029	
AG	0.60	0.28	4.70	0.030	1.82
AA	0.25	0.13	3.60	0.058	1.28

Nagelkerke *R*^2^ = 0.009.

**Table 5:** Correlation haplotype analysis of four SNPs in *MC4R* gene in normal weight and obesity subjects.

Type name	Structure	Obesity/normal weight (%)	*P*
Block 1			
Hap1	GTCT	76.0/78.0	0.12
Hap2	ACAC	23.0/21.0	**0.01**
Hap3	ATCT	1.4/0.8	0.26

Four SNPs includes rs2331841, rs6567160, rs571312, and rs17782313.

**Table 6:** Associations of SNPs in *MC4R* gene with DBP in all subjects.

SNP variables	Genotype			*P* ^*∗*^	*P* ^#^	*P* ^Δ^
rs2331841	AA	AG	GG			
DBP (mmHg)	82.06 (78.70, 85.42)	82.92 (81.42, 84.41)	80.44 (79.34, 81.54)	0.023	0.033	0.059

rs6567160	CC	CT	TT			
DBP (mmHg)	81.40 (77.94, 84.86)	83.20 (81.70, 84.70)	80.36 (79.27, 81.45)	0.011	0.014	0.030

rs571312	AA	AC	CC			
DBP (mmHg)	81.88 (78.22, 85.54)	83.24 (81.72, 84.76)	80.36 (79.27, 81.45)	0.012	0.013	0.033

rs17782313	CC	CT	TT			
DBP (mmHg)	80.40 (79.32, 81.49)	83.15 (81.63, 84.68)	81.81 (78.34, 85.27)	0.020	0.021	0.047

rs12970134	AA	AG	GG			
DBP (mmHg)	81.38 (76.48, 86.27)	82.86 (81.29, 84.42)	80.66 (79.60, 81.72)	0.033	0.034	0.151

rs11872992	AA	AG	GG			
DBP (mmHg)	81.46 (78.36, 84.24)	82.23 (81.01, 83.45)	80.98 (79.21, 82.68)	0.164	0.270	0.830

^*∗*^ANOVA (one-way analysis of variance) analysis between three genotypes. ^#^Comparison of the genotypes under the dominant model. ^Δ^Logistic regression analysis between three genotypes and DBP after adjusting for sex, age, and BMI. DBP: diastolic blood pressure.

## Data Availability

The datasets generated during and/or analyzed during the current study are available from the corresponding author on reasonable request.

## Additional Points

*Limitations.* The current study is a cross-sectional, small-sample study. A prospective cohort study is required to verify the association between *MC4R* polymorphisms and obesity and its related metabolic disorders. Further efforts should also be undertaken at the molecular and cellular levels to investigate the mechanism of the *MC4R* gene on obesity.

## Supplementary Materials

Supplementary Materials The supplementary material included supplementary tables for the study. Table S1: call rate and MAF in CHB for each SNP. Table S2: Hardy–Weinberg equilibrium test for each SNP. Table S3: associations of SNPs with anthropometric and metabolic parameters in all subjects.
