# Supplementary material for: MC4R Single Nucleotide Polymorphisms Were Associated with Metabolically Healthy and Unhealthy Obesity in Chinese Northern Han Populations
Source: Int J Endocrinol. 2019 Nov 6;2019:4328909. doi: 10.1155/2019/4328909 (PMC6875380; doi:10.1155/2019/4328909)
Supplement: Supplementary Materials — The supplementary material included supplementary tables for the study. Table S1: call rate and MAF in CHB for each SNP. Table S2: Hardy–Weinberg equilibrium test for each SNP. Table S3: associations of SNPs with anthropometric and metabolic parameters in all subjects. [file 4328909.f1.docx]

**Table S1 Call rate and MAF in CHB for each SNP**

| ***MC4R*** | **SNPs** | **Allelic gene** | **Call rate** | **MAF in CHB** | **MAF** |
| --- | --- | --- | --- | --- | --- |
|  | rs2331841 | A/G | 99.6% | 0.25 | 0.23 |
|  | rs6567160 | C/T | 99.8% | 0.19 | 0.22 |
|  | rs571312 | A/C | 99.2% | 0.14 | 0.21 |
|  | rs17782313 | C/T | 99.5% | 0.14 | 0.22 |
|  | rs12970134 | A/G | 99.5% | 0.14 | 0.20 |
|  | rs11872992 | A/G | 99.7% | 0.21 | 0.21 |

CHB：Han Chinese in Beijing,

**Table S2 Hardy-Weinberg Equilibrium test for each SNP**

| **SNPs** | **P** | | | |
| --- | --- | --- | --- | --- |
|  | MUH-NW | MH-NW | MUHO | MHO |
| rs2331841 | 0.54 | 0.56 | 1.00 | 1.00 |
| rs6567160 | 1.00 | 1.00 | 1.00 | 0.86 |
| rs571312 | 0.83 | 1.00 | 1.00 | 1.00 |
| rs17782313 | 1.00 | 0.66 | 0.86 | 0.85 |
| rs12970134 | 0.36 | 0.75 | 0.27 | 0.68 |
| rs11872992 | 0.16 | 0.12 | 0.39 | 0.37 |

MUH-NW, metabolic unhealthy normal weight;

MH-NW, metabolic healthy normal weight;

MUHO, metabolically unhealthy obesity;

MHO, metabolically healthy obesity

**Table S3 Associations of SNPs with anthropometric** **and metabolic parameters in all subjects**

| **rs17782313** | **CC** | **CT** | **TT** | **P** |
| --- | --- | --- | --- | --- |
| **BMI (kg/m2)** | 26.86(26.40,27.31) | 27.57(26.97,28.16) | 27.00(25.46,28.54) | 0.31 |
| **Weight(kg)** | 72.57(71.14,74.01) | 74.43(72.62,76.25) | 74.19(69.40,78.98) | 0.31 |
| **SBP(mmHg)** | 125.56(123.87,127.26) | 127.99(125.56,130.42) | 124.61(119.00,130.22) | 0.37 |
| **FBG（mmol/L）** | 5.70(5.59,5.82) | 5.74(5.55,5.93) | 5.66(5.32,6.00) | 0.43 |
| **TG（mmol/L）** | 2.09(1.93,2.25) | 1.85(1.69,2.02) | 1.71(1.39,2.03) | 0.20 |
| **HDL（mmol/L）** | 1.33(1.28,1.39) | 1.30(1.25,1.35) | 1.21(1.11,1.31) | 0.26 |
| **TC （mmol/L）** | 5.02(4.92,5.11) | 4.98(4.87,5.09) | 4.91(4.52,5.29) | 0.41 |
| **LDL（mmol/L）** | 3.16(3.09,3.24) | 3.13(3.04,3.23) | 3.13(2.83,3.42) | 0.49 |
| **rs571312** | AA | AC | CC | P |
| **BMI (kg/m2)** | 26.97(25.32,28.62) | 27.55(26.96,28.14) | 26.84(26.38,27.29) | 0.30 |
| **Weight(kg)** | 75.06(69.92,80.20) | 74.23(72.43,76.04) | 72.57(71.14,74.01) | 0.30 |
| **SBP(mmHg)** | 123.61(118.01,129.20) | 128.31(125.89,130.74) | 125.43(123.73,127.13) | 0.27 |
| **FBG（mmol/L）** | 5.71(5.34,6.07) | 5.75(5.56,5.94) | 5.70(5.58,5.82_ | 0.26 |
| **TG（mmol/L）** | 1.77(1.43,2.11) | 1.86(1.70,2.03) | 2.09(1.93,2.26) | 0.17 |
| **HDL（mmol/L）** | 1.19(1.09,1.28) | 1.29(1.24,1.35) | 1.33(1.28,1.39) | 0.26 |
| **TC （mmol/L）** | 4.78(4.48,5.09) | 5.01(4.89,5.12) | 5.01(4.91,5.11) | 0.36 |
| **LDL（mmol/L）** | 3.03(2.79,3.26) | 3.16(3.06,3.26) | 3.16(3.09,3.23) | 0.40 |
| **rs12970134** | AA | AG | GG | P |
| **BMI (kg/m2)** | 27.25(25.15,29.35) | 27.56(26.95,28.18) | 26.88(26.44,27.31) | 0.48 |
| **Weight(kg)** | 26.88(26.44,27.31) | 74.66(72.75,76.56) | 72.54(71.17,73.92) | 0.40 |
| **SBP(mmHg)** | 123.08(115.39,130.78) | 127.67(125.21,130.12) | 125.81(124.14,127.47) | 0.53 |
| **FBG（mmol/L）** | 5.63(5.20,6.05) | 5.73(5.54,5.92) | 5.71(5.60,5.83) | 0.37 |
| **TG（mmol/L）** | 1.64(1.33,1.96) | 1.82(1.67,1.97) | 2.09(1.93,2.26) | 0.25 |
| **HDL（mmol/L）** | 1.23(1.11,1.35) | 1.29(1.23,1.35) | 1.33(1.28,1.38) | 0.31 |
| **TC （mmol/L）** | 4.93(4.59,5.27) | 4.95(4.83,5.07) | 5.02(4.93,5.12) | 0.53 |
| **LDL（mmol/L）** | 3.13(2.87,3.38) | 3.13(3.03,3.23) | 3.17(3.09,3.24) | 0.50 |
| **rs6567160** | CC | CT | TT | P |
| **BMI (kg/m2)** | 27.26(25.64,28.88) | 27.54(26.95,28.12) | 26.84(26.38,27.29) | 0.23 |
| **Weight(kg)** | 75.20(70.13,80.27) | 74.24(72.46,76.03) | 72.57(71.14,74.01) | 0.25 |
| **SBP(mmHg)** | 123.29(117.88,128.69) | 128.19(125.80,130.59) | 125.43(123.73,127.13) | 0.27 |
| **FBG（mmol/L）** | 5.64(5.29,6.00) | 5.75(5.56,5.93) | 5.70(5.58,5.82) | 0.43 |
| **TG（mmol/L）** | 1.74(1.41,2.08) | 1.85(1.69,2.01) | 2.09(1.93,2.26) | 0.18 |
| **HDL（mmol/L）** | 1.21(1.12,1.31) | 1.30(1.25,1.35) | 1.33(1.28,1.39) | 0.38 |
| **TC （mmol/L）** | 4.79(4.50,5.08) | 5.00(4.89,5.12) | 5.01(4.91,5.11) | 0.39 |
| **LDL（mmol/L）** | 3.01(2.78,3.24) | 3.16(3.06,3.26) | 3.16(3.09,3.23) | 0.35 |
| **rs2331841** | AA | AG | GG | P |
| **BMI (kg/m2)** | 27.17(25.58,28.75) | 27.54(26.95,28.12) | 26.86(26.40,27.31) | 0.20 |
| **Weight(kg)** | 76.03(71.01,81.05) | 74.04(72.26,75.83) | 72.65(71.21,74.09) | 0.10 |
| **SBP(mmHg)** | 123.61(118.21,129.01) | 128.06(125.67,130.44) | 125.48(123.78,127.19) | 0.5 |
| **FBG（mmol/L）** | 5.64(5.30,5.98) | 5.77(5.58,5.95) | 5.69(5.57,5.80) | 0.47 |
| **TG（mmol/L）** | 1.84(1.52,2.15) | 1.86(1.70,2.02) | 2.09(1.92,2.25) | 0.15 |
| **HDL（mmol/L）** | 1.20(1.10,1.29) | 1.30(1.24,1.35) | 1.34(1.28,1.39) | 0.21 |
| **TC （mmol/L）** | 4.78(4.51,5.06) | 4.98(4.86,5.09) | 5.02(4.93,5.12) | 0.25 |
| **LDL（mmol/L）** | 3.02(2.80,3.24) | 3.14(3.05,3.24) | 3.17(3.09,3.24) | 0.36 |
| **rs11872992** | AA | AG | GG | P |
| **BMI (kg/m2)** | 27.26(25.83,28.70) | 27.05(26.45,27.66) | 27.12(26.67,27.57) | 0.23 |
| **Weight(kg)** | 75.29(71.25,79.33) | 72.32(70.47,74.17) | 73.58(72.15,75.00) | 0.20 |
| **SBP(mmHg)** | 124.13(117.26,131) | 125.45(123.18,127.72) | 126.97(125.24,128.70) | 0.94 |
| **DBP (mmHg)** | 81.16(76.55,85.76) | 81.04(79.54,82.54) | 81.64(80.56,82.73) | 0.96 |
| **FBG (mmol/L)** | 6.15(5.54,6.76) | 5.70(5.53,5.86) | 5.69(5.56,5.81) | 0.12 |
| **TG（mmol/L）** | 2.30(1.57,3.03) | 2.02(1.80,2.24) | 1.96(1.82,2.09) | 0.73 |
| **HDL（mmol/L）** | 1.23(1.06,1.40) | 1.38(1.30,1.47) | 1.29(1.25,1.33) | 0.26 |
| **TC （mmol/L）** | 4.96(4.71,5.22) | 5.10(4.97,5.24) | 4.95(4.86,5.04) | 0.69 |
| **LDL（mmol/L）** | 3.17(2.93,3.41) | 3.24(3.13,3.34) | 3.11(3.04,3.18) | 0.66 |
